# BLACK/WHITE DIFFERENCES IN LENGTH OF STAY IN HOSPICE AMONG MIDDLE-AGED AND OLDER ADULTS IN THE UNITED STATES

**DOI:** 10.1093/geroni/igad104.3012

**Published:** 2023-12-21

**Authors:** Heshuo Yu, J Scott Brown

**Affiliations:** Miami University in Oxford, Oxford, Ohio, United States; Miami University, Oxford, Ohio, United States

## Abstract

**Objectives:**

Hospice length of stay (LOS) is a crucial measure of hospice care. This topic is understudied within a nationally-representative sample across different races/ethnicities, especially among non-White decedents.

**Methods:**

The study used negative binomial regression to analyze three waves (2014, 2016, and 2018) from HRS (n=1,991).

**Results:**

African Americans were found to stay longer in hospice than their White counterparts when controlling for all other variables in the model. Not married, having Medicare insurance, cancer, chronic lung disease, and dementia significantly influence length of hospice stay when holding all other variables constant in the model.

**Discussion:**

The significant Black/White differences in hospice length of stay demonstrate the potential decrease in cumulative inequality in the delivery of appropriate and needed hospice services across the two racial groups. Future research is recommended to explore the potential consequences of longer hospice stays as well as the health and health care mechanisms related to the different disease profiles that lead individuals to hospice care, especially among African Americans.

